# Application of Neonatologist Performed Echocardiography in the assessment and management of persistent pulmonary hypertension of the newborn

**DOI:** 10.1038/s41390-018-0082-0

**Published:** 2018-08-02

**Authors:** Willem P. de Boode, Yogen Singh, Zoltan Molnar, Ulf Schubert, Marilena Savoia, Arvind Sehgal, Philip T. Levy, Patrick J. McNamara, Afif El-Khuffash, on behalf of the European Special Interest Group ‘Neonatologist Performed Echocardiography’ (NPE), T. Austin, K. Bohlin, M. C. Bravo, C. R. Breatnach, M. Breindahl, E. Dempsey, A. M. Groves, S. Gupta, B. Horsberg Eriksen, E. Nestaas, S. R. Rogerson, C. C. Roehr, C. E. Schwarz, M. G. Slieker, C. Tissot, R. van der Lee, D. van Laere, B. van Overmeire, L. van Wyk

**Affiliations:** 1 Department of Neonatology, Radboud University Medical Center, Radboud Institute for Health Sciences, Amalia Children’s Hospital, Nijmegen, The Netherlands; 2 Addenbrooke′s Hospital, Cambridge University Hospitals NHS Foundation Trust, Cambridge, United Kingdom; 3 John Radcliffe Hospital, Oxford, United Kingdom; 4 Department of Clinical Science, Intervention and Technology, Karolinska Institutet, Stockholm, Sweden; 5 Azienda Ospedaliero-Universitaria S. Maria della Misericordia, Udine, Italy; 6 Department of Pediatrics, Monash University, Melbourne, Australia; 7 Department of Pediatrics, Washington University School of Medicine, Saint Louis, MO USA; 8 Department of Pediatrics, Goryeb Children’s Hospital, Morristown, NJ USA; 9 Departments of Pediatrics and Physiology, University of Toronto, Toronto, ON Canada; 10 Department of Neonatology, The Rotunda Hospital, Dublin, Ireland; 11 Department of Pediatrics, The Royal College of Surgeons in Ireland, Dublin, Ireland; 12 Department of Neonatology, Rosie Hospital, Cambridge University Hospitals NHS Foundation Trust, Cambridge, United Kingdom; 13 Department of Neonatology, Karolinska University Hospital, Karolinska Institutet, Stockholm, Sweden; 14 Department of Neonatology, La Paz University Hospital, Madrid, Spain; 15 Department of Neonatology, The Rotunda Hospital, Dublin, Ireland; 16 Karolinska University Hospital, Karolinska Institutet, Stockholm, Sweden; 17 INFANT Centre, Cork University Maternity Hospital, University College, Cork, Ireland; 18 Division of Newborn Medicine, Mount Sinai Kravis Children’s Hospital, New York, NY USA; 19 University Hospital of North Tees, Durham University, Stockton-on-Tees, United Kingdom; 20 Department of Pediatrics, Møre and Romsdal Hospital Trust, Ålesund, Norway; 21 Institute of Clinical Medicine, Faculty of Medicine, University of, Oslo, Norway; 22 Department of Cardiology and Center for Cardiological Innovation, Oslo University Hospital, Rikshospitalet, Oslo, Norway; 23 Department of Paediatrics, Vestfold Hospital Trust, Tønsberg, Norway; 24 The Royal Women′s Hospital, Parkville, VIC Australia; 25 Department of Paediatrics, University of Oxford, John Radcliffe Hospital, Oxford, United Kingdom; 26 Department of Neonatology, University Children’s Hospital of Tübingen, Tübingen, Germany; 27 Department of Paediatric Cardiology, Radboudumc Amalia Children’s Hospital, Nijmegen, The Netherlands; 28 Department of Pediatrics, Clinique des Grangettes, Chêne Bougeries, Switzerland; 29 Department of Neonatology, Radboud university medical center, Radboud Institute for Health Sciences, Amalia Children’s Hospital, Nijmegen, The Netherlands; 30 Department of Pediatrics, Antwerp University Hospital UZA, Edegem, Belgium; 31 Department of Neonatology, University Hospital Brussels, Brussels, Belgium; 32 Department of Paediatrics & Child Health, University of Stellenbosch, Cape Town, South Africa

## Abstract

Pulmonary hypertension contributes to morbidity and mortality in both the term newborn infant, referred to as persistent pulmonary hypertension of the newborn (PPHN), and the premature infant, in the setting of abnormal pulmonary vasculature development and arrested growth. In the term infant, PPHN is characterized by the failure of the physiological postnatal decrease in pulmonary vascular resistance that results in impaired oxygenation, right ventricular failure, and pulmonary-to-systemic shunting. The pulmonary vasculature is either maladapted, maldeveloped, or underdeveloped. In the premature infant, the mechanisms are similar in that the early onset pulmonary hypertension (PH) is due to pulmonary vascular immaturity and its underdevelopment, while late onset PH is due to the maladaptation of the pulmonary circulation that is seen with severe bronchopulmonary dysplasia. This may lead to cor-pulmonale if left undiagnosed and untreated. Neonatologist performed echocardiography (NPE) should be considered in any preterm or term neonate that presents with risk factors suggesting PPHN. In this review, we discuss the risk factors for PPHN in term and preterm infants, the etiologies, and the pathophysiological mechanisms as they relate to growth and development of the pulmonary vasculature. We explore the applications of NPE techniques that aid in the correct diagnostic and pathophysiological assessment of the most common neonatal etiologies of PPHN and provide guidelines for using these techniques to optimize the management of the neonate with PPHN.

## Introduction

Persistent pulmonary hypertension of the newborn (PPHN) is a complex disorder that is characterized by the presence of an increased pulmonary vascular resistance (PVR) associated with shunting of deoxygenated blood from the pulmonary to the systemic circulation causing severe hypoxemia.

PPHN occurs in about 1–2 per thousand live born infants, mostly in term and late preterm newborns.^1–4^ It is associated with an increased risk of an adverse outcome (5-year survival approximately 90%; neurologic impairment in 15–25%) ^4,5^. The vascular pruning, abnormal vasculature, and vaso-reactivity in infants with bronchopulmonary dysplasia (BPD) set the tone for the development of pulmonary hypertension (PH) in this population as well.^6–8^ It is a known complication of BPD, with the incidence increasing with the severity of BPD.^9^ In infants with ‘severe’ BPD, two recent cohorts put the incidence at >50%.^9,10^ Echocardiographic signs of PH in preterm infants in an early phase (72 h to 14 days of age) are associated with decreased in-hospital survival and an increased incidence of moderate-severe BPD.^11^

Common risk factors for the development of PPHN in (near-) term infants and PH in preterm neonates are summarized in Table 1.^7,12,13^

**Table 1:** Risk factors for pulmonary hypertension in (near-) term and preterm infants

Term and near-term infants (PPHN)	Preterm infants (PH)
• Male gender • African or Asian maternal race • Maternal morbidity, such as obesity, diabetes, and asthma • Birth after cesarean section • Chorioamnionitis • Meconium-stained amniotic fluid • Antenatal exposure to selective serotonin re-uptake inhibitors (SSRI), cyclooxygenase inhibitors (COXi), certain “medications” (Chinese herbs) • Perinatal infection • Perinatal asphyxia • Hypothermia • Metabolic derangements, like hypocalcaemia and acidosis • Stress, pain stimuli • Polycythemia • Trisomy 21	• Severe bronchopulmonary dysplasia • Lower gestational age at birth • Lower birth weight • Small for gestational age • Pulmonary hemorrhage • Sepsis • Oligohydramnios and anhydramnios • Prolonged duration of invasive respiratory support • Increased length of stay in hospital

The typical clinical picture is a patient with hypoxic cardio-respiratory failure with a pre-/postductal difference in oxygen saturation of ≥5%, although this difference will be attenuated in the presence of a relevant atrial right-to-left shunt. It should be noted that a pre-/postductal oxygen saturation difference can also be caused by left-sided obstructive heart disease, such as coarctation of aorta, interrupted aortic arch, and hypoplastic left heart disease. Measuring pre- and postductal blood pressure can also be helpful and may give insights regarding the presence of shunts. Postductal blood pressure may be heavily influenced by a right-to-left transductal shunt and falsely miss low preductal blood pressure, which is crucial.

Distinguishing between PPHN and cyanotic heart lesions on clinical grounds can be challenging. This, however, is essential since management for these disorders are quite different. Clinical signs and symptoms suggestive of an underlying congenital heart defect are summarized in Table 2.

**Table 2:** Clinical signs and symptoms suggestive for a congenital heart defect (‘red flags’)

• Absence of suggestive risk factors or clinical triggers (Table 1)
• No signs of respiratory distress
• Presence of heart murmur
• Reduced femoral pulsations
• Abnormal heart configuration/cardiomegaly on chest X-ray
• Blood pressure gradient between upper and lower body
• Lack of response to high concentrations of oxygen
• No effect of nitric oxide inhalation or other vasodilators

Delay in the diagnosis and appropriate treatment of a cyanotic heart disease is associated with worsening prognosis. It is therefore imperative to have a comprehensive echocardiographic evaluation to rule out any structural abnormality. When in doubt during the time before echocardiography can be performed, it is advised to start prostaglandin E1 in anticipation of a potential heart defect. In a patient with PPHN, prostaglandin E1 might have some pulmonary vasodilatory effects, and in severe PPHN, it will preserve postductal systemic perfusion, albeit at the expense of cyanosis. It should be noted that the institution of a treatment regimen aiming at pulmonary vasodilation may deteriorate the clinical condition of the patients with certain heart defects, such as total anomalous pulmonary venous connection (TAPVC) and hypoplastic left heart syndrome.

The following structural heart defects may clinically mimic PPHN: TAPVC, transposition of great arteries (TGA), pulmonary atresia with or without ventricular septal defect (VSD), severe Fallot’s tetralogy, tricuspid atresia, unguarded tricuspid orifice syndrome, severe Ebstein anomaly, and sometimes even left-sided obstructive heart disease (such as coarctation of aorta, interrupted aortic arch, and hypoplastic left heart disease).

## Etiology

Pulmonary artery pressure (PAP) is determined by pulmonary blood flow (PBF), PVR, and pulmonary capillary wedge pressure (PcWP), as shown in the following Eq. 1.

1 $$\hskip 70pt{\mathrm{PAP}}\,{\mathrm{ = }}\,{\mathrm{PcWP}}\,{\mathrm{ + }}\,\left( {{\mathrm{PBF}}\,\,\times\,{\mathrm{PVR}}} \right).$$

Under normal circumstances, PAP falls after birth within 2 months to reach a level that is comparable to adult values (systolic PAP <25 mmHg).

As can be derived from Eq. 1, PH can be caused by an increase in PcWP (left heart failure, for example secondary to arteriovenous malformations, such as vein of Galen aneurysmal malformation (VGAM)), by an increase in PBF (e.g., large left-to-right shunt with pulmonary hyper-perfusion), or by raised PVR (e.g., pulmonary vasoconstriction). A combination of these factors is also possible.

In PPHN, the rise in pulmonary blood pressure is generally secondary to an increased PVR with the following etiology:^14^

1. Maladaption of pulmonary vasculature (abnormal, ‘reactive’ pulmonary vasoconstriction)
  1.1. due to parenchymal lung diseases, such as meconium aspiration syndrome (MAS), respiratory distress syndrome (RDS), hypoventilation, and pneumonia
  1.2. in response to certain stimuli, such as hypothermia, sepsis, stress, hypercapnia, hypoxemia, acidosis, and hyperviscosity
  1.3. toxic/pharmacological (maternal SSRI use)
2. Maldevelopment of pulmonary vasculature (remodeling of pulmonary vasculature) in response to:
  2.1. in utero closure of ductus arteriosus (for example maternal cyclooxygenase inhibitor use)
  2.2. pulmonary hyperperfusion in congenital heart disease with large left-to-right shunt
  2.3. infants with fetal growth restriction
3. Underdevelopment of pulmonary vasculature (hypoplastic pulmonary vessels; decreased cross-sectional area), such as in:
  3.1. congenital diaphragmatic hernia
  3.2. pulmonary hypoplasia (premature prolonged rupture of membranes, oligohydramnios and anhydramnios).

Respiratory disorders that are associated with PH include: congenital diaphragmatic hernia, BPD, alveolar capillary dysplasia (with or without misalignment of veins), lung hypoplasia (‘primary’ or ‘secondary’), surfactant protein abnormalities, pulmonary interstitial glycogenosis, pulmonary alveolar proteinosis, and pulmonary lymphangiectasis.^15^ Recently, it was recognized that PH can be caused by pulmonary venous stenosis, especially in preterm infants with BPD, that is often overlooked.^16,17^ In about 10–20%, no specific cause of PPHN is found (‘idiopathic’ PPHN).^14^

## Hemodynamic profile of pphn

The key features in PPHN are an increased PVR resulting in high PAP, ductal and/or atrial right-to-left shunting, and right (and ultimately left) ventricular dysfunction. This leads to:

• Right ventricular systolic and diastolic failure secondary to increased afterload
• Decrease in RV stroke volume and RV filling
• Decrease in pulmonary blood flow with ventilation-perfusion mismatch
• RV dilatation causing a D-shaped left ventricle with decreased LV preload
• Decreased LV stroke volume
• Right-to-left shunting through the ductus arteriosus and/or foramen ovale.

In a severe form of PPHN, this ductal and/or atrial right-to-left shunt may guarantee postductal or preductal systemic perfusion, respectively, however, at the expense of cyanosis.

## Echocardiography

Comprehensive echocardiography is indicated when there is a clinical suspicion of PPHN to exclude congenital heart disease.

Neonatologist performed echocardiography (NPE) is useful in multiple ways: (a) making the diagnosis and grading the severity, (b) determining the need for specific (pulmonary vasodilator) or supportive (choice of inotrope) therapy, (c) monitoring the response to therapy, and (d) rational weaning of therapy. This is especially relevant when administering inhaled nitric oxide (iNO) for infants <34 weeks’ gestation, where multiple randomized controlled trials  have suggested limited evidence.^18,19^ According to the recent AHA/ATS guidelines, iNO can be beneficial for preterm infants with severe hypoxemia that is due primarily to PPHN physiology rather than parenchymal lung disease, particularly if associated with prolonged rupture of membranes and oligohydramnios.^20^

Once the diagnosis of PPHN is confirmed by echocardiography, the clinical course and the effects of medical interventions can be monitored using NPE with the emphasis on:

1. pulmonary artery pressure and PVR,

2. myocardial performance, and

3. shunting through ductus arteriosus and open foramen ovale.

An overview of all echocardiographic parameters that can be assessed with NPE is presented in Table 3.

**Table 3:** Overview of echocardiographic parameters for the assessment of pulmonary artery pressure (PAP), pulmonary vascular resistance (PVR), right ventricular (RV) performance, and shunts in patients with PPHN

PAP	PVR	RV performance	Shunts
1. TR peak velocity (SPAP) 2. PI peak velocity (DPAP) 3. Transductal RtL flow peak velocity (SPAP) 4. IVS configuration/LV-sEI	5. RV systolic time intervals PAAT PAAT/RVET ratio RVPET/RVET ratio 6. TRV/VTI^[RVOT]^ ratio 7. Pulmonary artery compliance	8. TAPSE 9. FAC 10. MPI 11. RV S/D ratio 12. TDI RV free wall & IVS 13. STE	14. Transductal shunting Direction Peak flow velocity 15. Interatrial shunting Direction

### Estimation of PAP or PVR

PAP can be assessed by measuring tricuspid valve regurgitation peak velocity, pulmonary regurgitation peak velocity, transductal right-to-left flow peak velocity, interventricular septum (IVS) configuration, and LV systolic eccentricity index (LV-sEI).

#### Tricuspid regurgitation peak velocity

Systolic pulmonary artery pressure (SPAP) can be estimated by measuring the peak velocity of tricuspid valve regurgitation with the use of the modified Bernoulli’s equation; see Eqs. 2 and 3:

2 $$\begin{array}{l}p = 4\,\times\,{v}^2\\ \quad \,\hskip -14pt\left({p,{\rm{pressure}}\,{\rm{gradient}}\ \left( {{\rm{in}}\,{\rm{mmHg}}} \right);\,v,{\rm{blood}}\,{\rm{velocity}}\,\left({{\rm{in}}\,{\rm{m}}/{\rm{s}}} \right)} \right),\end{array}$$

3 $$\begin{array}{l}{\mathrm{SPAP}} \approx {\mathrm{RVSP}} = {\mathrm{4}}\,\times\left( {{\mathrm{VmaxTR}}} \right)^{\mathrm{2}} + {\mathrm{RAP}}\\ \quad \left( {{\rm{RSVP}},{\rm{right}}\,{\rm{ventricular}}\,{\rm{systolic}}\,{\rm{pressure}}\,\left({{\rm{in}}\,{\rm{mmHg}}}\right);} \right.\\ \quad {\rm{VmaxTR}},{\rm{peak}}\,{\rm{velocity}}\,{\rm{of}}\,{\rm{tricuspid}}\,{\rm{regurgitation}}\,\left({{\rm{in}}\,{\rm{m}}{\mathrm{/}}{\rm{s}}} \right);\\ \quad \left. {{\rm{RAP}},{\rm{right}}\,{\rm{atrial}}\,{\rm{pressure}}\,\left( {{\rm{in}}\,{\rm{mmHg}}}\right)}\right)\end{array}$$

**Fig. 1:**
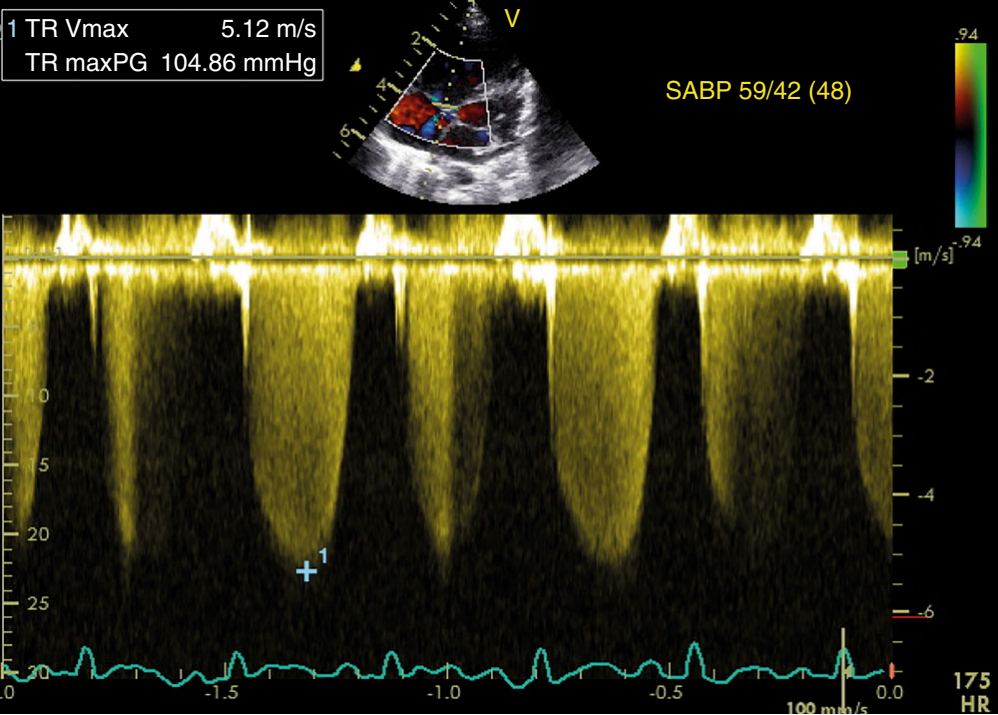
Estimation of SPAP from tricuspid regurgitation jet. The peak velocity of the tricuspid regurgitation jet is 5.12 m/s, which corresponds to a maximum pressure gradient of 105 mmHg according to the modified Bernoulli equation (see left upper panel). On the right, the concomitant systemic arterial blood pressure is displayed, indicating suprasystemic pulmonary pressure

RAP is usually not measured, and a value of 3–5 mmHg is generally assumed. The estimation of SPAP by measuring TR is reliable and often equivalent to pressures measured in the catheter lab while using continuous wave Doppler (Fig. 1). However, the accuracy depends on the quality of the acquired TR jet. An optimal quality TR jet shows a well demarcated envelope. Measuring an inadequate Doppler spectral envelope will potentially lead to an underestimation of SPAP. The angle of insonation should be less than 20° to achieve a reliable measurement. This is aided by assessing maximal TR jet velocity by imaging from three views (apical 4 chamber, short axis, and modified parasternal long axis). However, this estimation of SPAP is not reliable in the presence of right ventricular failure or right ventricular outflow tract obstruction. Tricuspid valve regurgitation cannot always be observed and is present in approximately 60–85% of patients with PPHN.^21–25^

#### Pulmonary regurgitation peak velocity

In the presence of pulmonary valve regurgitation, mean PAP (MPAP) can be estimated by measuring its peak velocity using Eq. 4:

4 $$\begin{array}{l}{\mathrm{MPAP}} = {\mathrm{4}}\,\times\left( {{\mathrm{VmaxPR}}} \right)^{\mathrm{2}} +\, {\mathrm{RVdP}}\\ \quad \left( {{\mathrm{MPAP}},{\mathrm{mean}}\,{\mathrm{pulmonary}}\,{\mathrm{artery}}\,{\mathrm{pressure}}\,\left( {{\mathrm{in}}\,{\mathrm{mmHg}}} \right);} \right.\\ \quad {\mathrm{VmaxPR}},{\mathrm{peak}}\,{\mathrm{velocity}}\,{\mathrm{pulmonary}}\,{\mathrm{valve}}\,{\mathrm{incompetence}}\,\left( {{\mathrm{in}}\,{\mathrm{m}}{\mathrm{/}}{\mathrm{s}}} \right);\\ \quad \left. {{\mathrm{RVdP}},\,{\mathrm{right}}\,{\mathrm{ventricular}}\,{\mathrm{diastolic}}\,{\mathrm{pressure}}\,\left( {{\mathrm{in}}\,{\mathrm{mmHg}}} \right)} \right).\end{array}$$

The RVdP is generally assumed to be around 2–5 mmHg.

#### Transductal right-to-left blood flow peak velocity

Transductal right-to-left blood flow can be used to estimate SPAP, when it lasts ≥30% of the heart cycle, by measuring its peak velocity using Eq. 5:

5 $$\begin{array}{l}{\mathrm{SPAP}} = {\mathrm{4}}\,{\mathrm{x}}\,\left( {{\mathrm{VmaxDA}}} \right)^{\mathrm{2}}\, + \,{\mathrm{SSAP}}\\ \quad \left( {{\mathrm{SPAP}},{\mathrm{systolic}}\,{\mathrm{pulmonary}}\,{\mathrm{artery}}\,{\mathrm{pressure}}\,\left( {{\mathrm{in}}\,{\mathrm{mmHg}}} \right);} \right.\\ \hskip -14pt\quad {\mathrm{VmaxDA}},{\mathrm{peak}}\,{\mathrm{velocity}}\,{\mathrm{ductal}}\,{\mathrm{right}}{\mathrm{ - }}{\mathrm{to}}{\mathrm{ - }}{\mathrm{left}}\,{\mathrm{shunt}}\,\left( {{\mathrm{in}}\,{\mathrm{m}}{\mathrm{/}}{\mathrm{s}}} \right);\\ \quad \left. {{\mathrm{SSAP}},{\mathrm{systolic}}\,{\mathrm{systemic}}\,{\mathrm{arterial}}\,{\mathrm{pressure}}\,\left( {{\mathrm{in}}\,{\mathrm{mmHg}}} \right)} \right).\end{array}$$

A ductal right-to-left or bidirectional shunt is observed in 73–91% of the patients with PPHN.^11,12,15^ However, measurement of PAP via ductal flow is often not reliable. Assessment of the direction of transductal blood flow is more useful and will indicate the relation between pulmonary and systemic pressures.

#### IVS configuration/LV-sEI

**Fig. 2:**
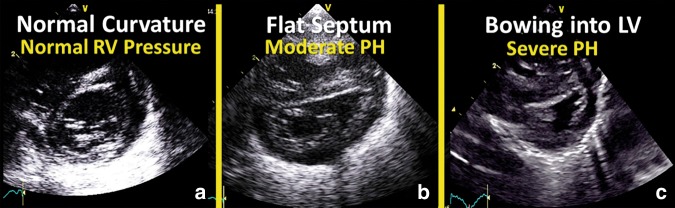
Morphology of interventricular septum. Normally the septum bows into the right ventricle (**a**; O-shaped LV) and with increasing right ventricular pressure, the interventricular septum will flatten (**b**; D-shaped LV) and eventually curves into the left ventricle (**c**; crescent-shaped LV)

An alternative, although more subjective, estimation of PAP is based upon the alignment of the IVS (Table 4). Normally the septum bows into the right ventricle (O-shaped LV) and with increasing right ventricular pressure, the IVS will flatten (D-shaped LV) and eventually curves into the left ventricle (crescent-shaped LV) (see Fig. 2).^5^ It is best analyzed at the end of systole in the parasternal short axis view above the level of the papillary muscles.^26^

**Table 4:** Estimation of RVP based on LV configuration

Left ventricular configuration	Estimated RVP
O-shaped LV	<50% of LVP
D-shaped LV	50–100% of LVP
Crescent-shaped LV	≥100% of LVP

*LV* left ventricle, *RVP* right ventricular pressure, *LVP* left ventricular pressure

**Fig. 3:**
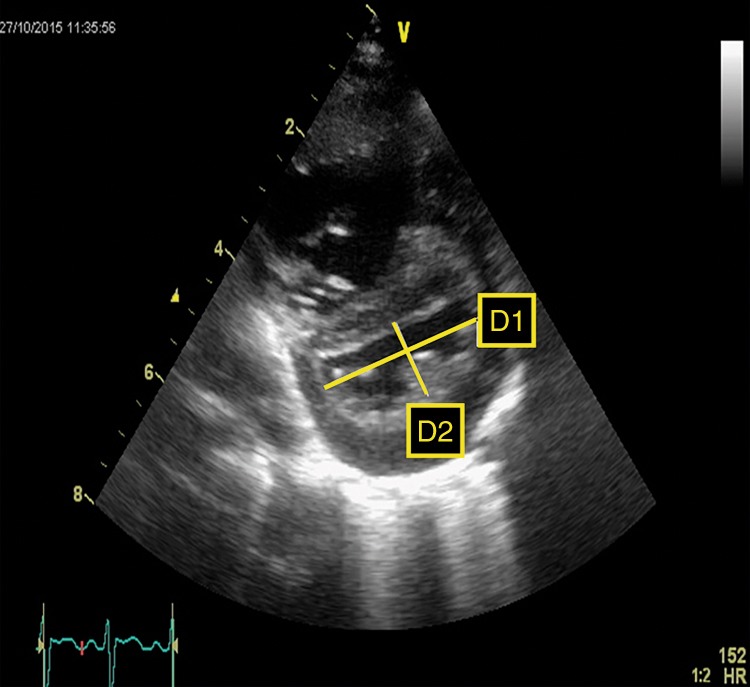
LV systolic eccentricity index (LV-sEI). Left ventricle from short axis view showing flattened septum and high systolic eccentricity index (LV-sEI = *D*1/*D*2)

A more objective estimation is made by calculating the LV-sEI, which is the ratio of LV dimension parallel and perpendicular to the septum, respectively. Figure 3 shows the measurement of LV-sEI from short axis parasternal view. The LV-sEI measure is a derivation from IVS configuration. Normal LV-sEI ratio is typically 1 and as it increases in PH, it allows for quantification of a more subjective parameter of IVS flattening/bowing. In adult literature, a ratio >1 in systole denotes RV pressure overload. In a recent study on infants with BPD associated PH, LV-sEI was significantly higher in the infants with PH.^27^ LV diastolic EI is more a marker of volume overloaded right ventricle; for clinical conditions such as PPHN, pressure overload predominates, and hence the usefulness of the sEI.

PVR can be assessed by measuring right ventricular systolic time intervals (pulmonary artery acceleration time (PAAT), right ventricular ejection time (RVET), right ventricular pre-ejection time (RVPET)), TRV:VTI^[RVOT]^-ratio (ratio between tricuspid regurgitation velocity (TRV) and the velocity–time integral (VTI) of blood flow through the right ventricular outflow tract (RVOT)), and pulmonary artery compliance.

#### Right ventricular systolic time intervals

**Fig. 4:**
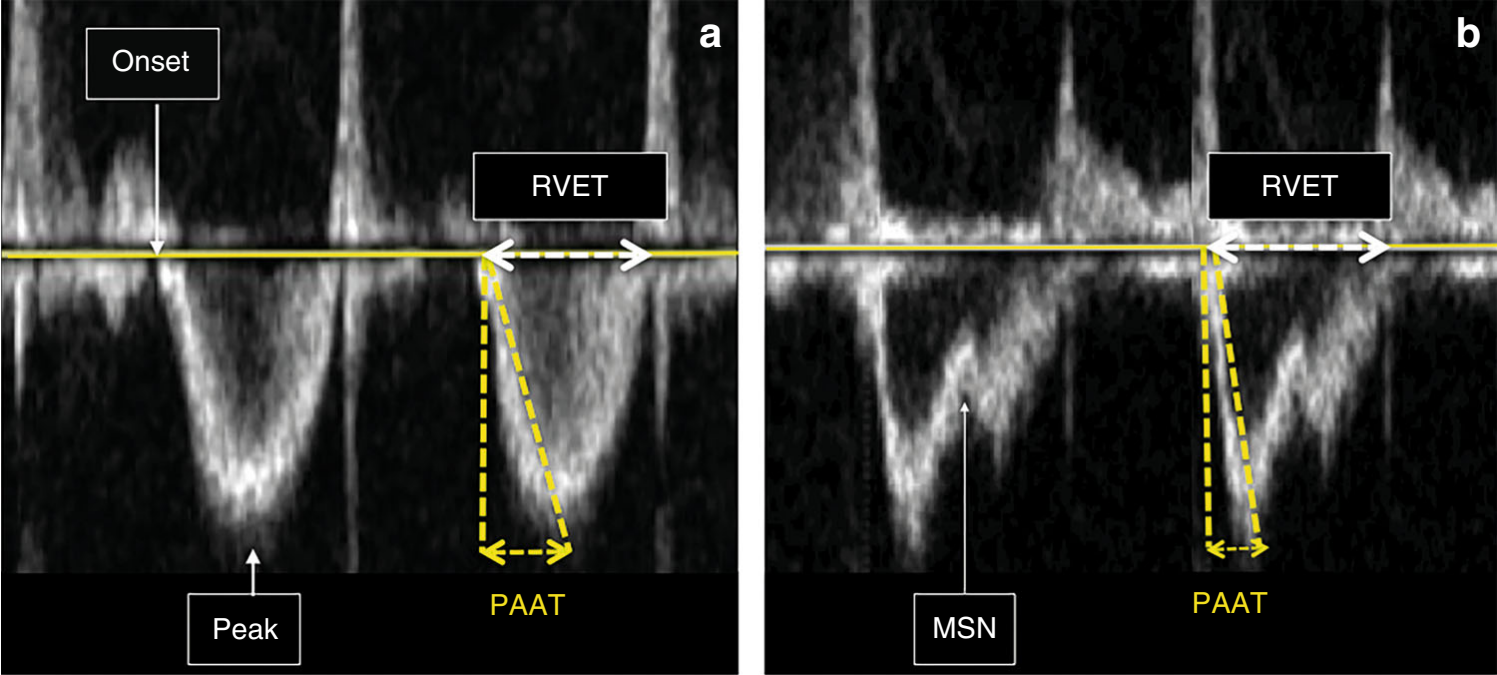
Right ventricular systolic time intervals. **a** Normal pulmonary artery pressure/pulmonary vascular resistance. **b** Increased pulmonary artery pressure/pulmonary vascular resistance (MSN mid-systolic notch, PAAT pulmonary artery acceleration time, peak peak velocity, RVET right ventricular ejection time). See text for details

Right ventricular systolic time intervals are another validated method for the estimation of PVR. The following right ventricular time intervals can be derived from the Doppler PBF velocity curve: RVET, RVPET, and PAAT, also referred to as time to peak velocity (TPV) (see Fig. 4).

Until recently, the methods for the estimation of PAP using the peak velocity of pulmonary regurgitation was considered more reliable than right ventricular systolic time intervals, since the repeatability of these time intervals was shown to be rather disappointing.^28–30^ However, PAAT has recently been validated as a feasible and reproducible, non-invasive echocardiographic imaging marker, for detection of pulmonary vascular disease and PH in neonates and children.^31^ This study, with simultaneous Doppler echocardiography and invasive catheterization, established PAAT-based regression equations in children to accurately predict invasive catheterization-derived SPAP and PVR. A cutoff value of <90 ms reliably detects pulmonary vascular disease, and a value <40 ms detects pulmonary vascular disease in its most severe form of PH. The normal value of the PAAT:RVET ratio is approximately 0.31 or greater. A PAAT:RVET ratio less than 0.23 and/or an increased RVPET:RVET ratio is indicative for increased PAP.^31^ Visual inspection of the shape of the Doppler flow envelope pattern across the RV outflow tract is a sensitive predictor of PH and right heart dysfunction in children and infants. The mid-systolic notch, also referred to as the “flying W”, is associated with elevated PVR and PAP (Fig. 4—right panel).^32^

#### TRV/VTI^[RVOT]^

PVR can be estimated by calculating the TRV:VTI^[RVOT]^ ratio, which is the ratio between TRV and the VTI of blood flow through the RVOT using pulsed-wave Doppler in the parasternal short axis.^33–35^ The obtained VTI in the RVOT will be markedly changed in the presence of high PVR due to an earlier and enhanced reflection of the pressure wave. Higher PVR will lead to a decrease in VTI^[RVOT]^. TRV:VTI^[RVOT]^ ratio have been shown to correlate well with PVR in children and a cut off value of 0.14 provided high predictive values.^34^ However, neonatal studies are lacking.

#### Pulmonary artery compliance

Echocardiography can be used to estimate dynamic pulmonary artery compliance (CdynPA) non-invasively by measuring pulmonary diameter in systole (Ds) and diastole (Dd) and analyzing the TR jet to calculate SPAP.^36^

6 $$\begin{array}{l}{\mathrm{CdynPA}} = \left[ {\left( {{\mathrm{Ds}} - {\mathrm{Dd}}} \right){\mathrm{/}}\left( {{\mathrm{Dd}} \times {\mathrm{SPAP}}} \right)} \right] \times {\mathrm{10}}^{\mathrm{4}}\\ {\mathrm{CdynPA}},{\mathrm{dynamic}}\,{\mathrm{pulmonary}}\,{\mathrm{artery}}\,{\mathrm{compliance}} \\ \left( {{\mathrm{in}}\,\% \,{\mathrm{change}}{\mathrm{/}}100\,{\mathrm{mmHg}}} \right) D,{\mathrm{diameter}}\,\left( {{\mathrm{in}}\,{\mathrm{cm}}} \right).\end{array}$$

Lower CdynPA is found in children with PH.^36^ There is a paucity of data on this measurement in the term and preterm neonatal population.

#### Qualitative assessment of right ventricular, right atrial, and pulmonary diameters

An increased PVR can cause an increased pulmonary artery diameter. Impaired right ventricular systolic and diastolic performance will lead to dilation of the right ventricle, right atrium, and inferior vena cava. Normative data for right ventricular size have been published.^37–40^

#### Myocardial performance

Biventricular dysfunction can be found in up to 70% of patients with PPHN and is thought to be related to increased right ventricular afterload, decreased left ventricular preload in addition to possible myocardial ischemia.^24,25^

Classical PPHN (acute, soon after birth, secondary to pathologies like MAS) is a pre-capillary disorder. The right heart is dilated but the LV is not (due to reduced preload to LV, and septal shift into the LV). One might find lower PcWP, as the malady is caused by elevated PVR. Hence, pulmonary vasodilators work well. BPD associated PH is another beast, and some infants have post-capillary pathophysiology as an important contributor. This group is clinically characterized by an elevated PcWP and no response to or deterioration after start of pulmonary vasodilators such as iNO and sildenafil. Unfortunately, invasive cardiac catheterization is not easily available, and many of these infants may not tolerate that. Echocardiographic clues to this malady are dilated LV and increased LA end-diastolic pressure. This cohort needs systemic and not pulmonary vasodilators as the pathophysiology centers on elevated ‘systemic’ more than ‘pulmonary afterload’.^41^

A nearly consistent finding in (near-)term patients with PPHN is a low left ventricular output (LVO) and reduced LV stroke volume (LV-SV) in combination with normal or mildly decreased LV-EF, that is explained by reduced preload secondary to right-to-left shunting (decreased pulmonary venous return) and ventricular–ventricular interaction (flattening of IVS).^21,22,25^

Echocardiographic markers of left ventricular failure (reduced LV size and stroke volume) are associated with the need for more intense treatment, such as high frequency ventilation and ECMO in newborn infants diagnosed with PPHN.^23^

Right ventricular function can be assessed by measuring the fractional area change (FAC), myocardial performance index (RV-MPI), right ventricular systolic to diastolic duration ratio (RV S/D ratio), and tricuspid annular plane systolic excursion (TAPSE).

Left ventricular function can be analyzed by monitoring myocardial performance (LV-MPI), LV-SV and LVO, and ejection fraction (EF biplane Simpson).

By using tissue Doppler imaging (TDI) and speckle-tracking echocardiography (STE), the performance of both ventricles can be assessed.

#### Tricuspid annular plane systolic excursion (TAPSE)

**Fig. 5:**
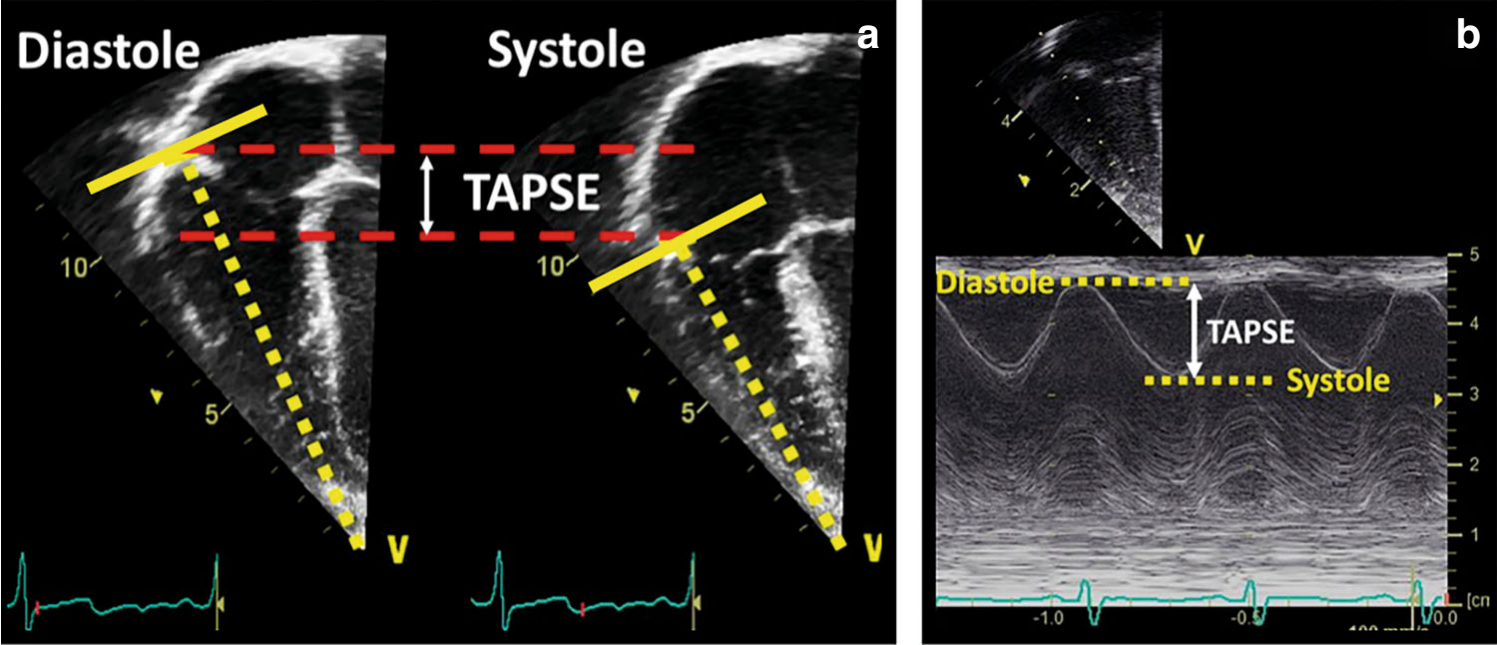
Tricuspid annular plane systolic excursion (TAPSE). TAPSE is a measure of RV longitudinal function and obtained from the 4-chamber view using the M-Mode with the cursor aligned along the direction of the lateral annulus. The traveled distance of the tricuspid annulus from diastole to systole is expressed in millimeters

TAPSE is a measure of RV longitudinal function and obtained from the 4-chamber view using the M-Mode with the cursor aligned along the direction of the lateral annulus (see Fig. 5). TAPSE provides useful information about longitudinal fiber shortening and it has shown good correlation with techniques estimating RV global systolic function. However, it should be noted that TAPSE is both angle and load dependent. Normal values in the neonatal population can be obtained from Koestenberger et al.^42^ Diminished TAPSE (<4 mm) is predictive for the need of ECMO and death in infants with PPHN.^43^

#### Fractional area change (FAC)

FAC is a planimetric measure of the ratio of systolic to diastolic area in apical 4- or 3-chamber view by manual tracing of the endocardial border of the right ventricle. Unlike TAPSE, right ventricular FAC is affected by radial, basal, and apical functions as well as longitudinal fiber shortening, but it is considered to be more prone to operator-dependent variation. FAC is calculated by the following formula:

7 $${\mathrm{FAC}} = \left[ {{\mathrm{RV}}\,{\mathrm{area}}\,\left( {{\mathrm{diastole}}} \right)-{\mathrm{RV}}\,{\mathrm{area}}\,\left( {{\mathrm{systole}}} \right)} \right]{\mathrm{/RV}}\,{\mathrm{area}}\,\left( {{\mathrm{diastole}}} \right).$$

**Fig. 6:**
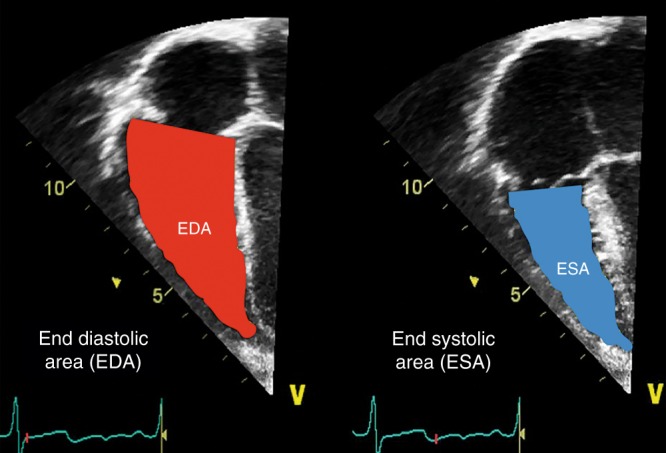
Fractional area change (FAC). FAC is a planimetric measure of the ratio of end systolic area (ESA) to end diastolic area (EDA) in apical 4- or 3-chamber view by manual tracing of the endocardial border of the right ventricle (FAC (%) = [EDA−ESA]/EDA). Trabeculation should be included within the cavity under the tracing procedure

It is important that the entire ventricle is visualized when tracing the endocardium in systole and diastole including the outflow tract and the lateral wall (Fig. 6). Trabeculation should be included within the cavity under the tracing procedure. Normal values (25–45%) in preterm and term infants have been published.^38,43,44^ Median values of 19% were associated with the need for ECMO or death.^43^

#### Myocardial Performance Index (MPI)

**Fig. 7:**
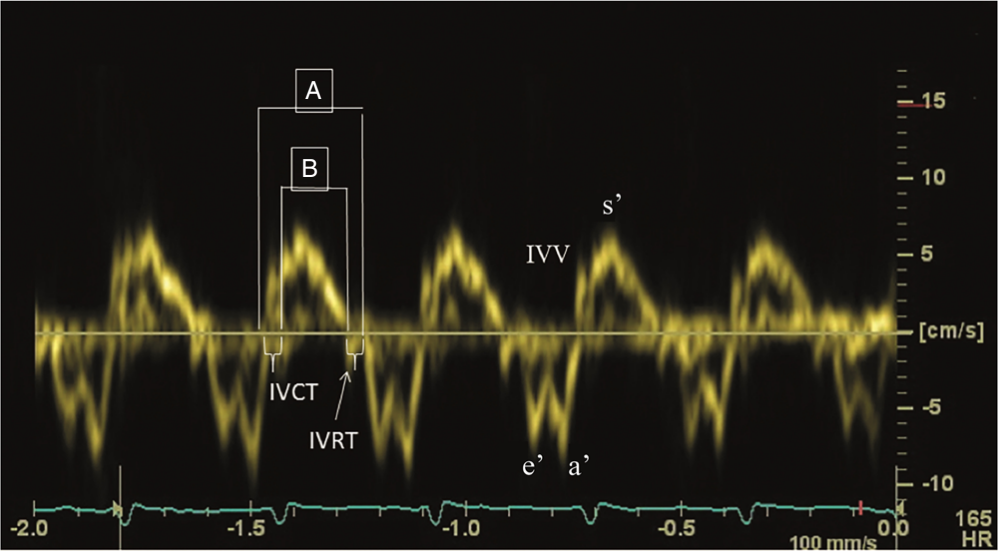
Myocardial Performance Index—MPI or Tei index. Pulse wave Tissue Doppler waveform (isovolumic contraction time (IVCT), isovolumic relaxation time (IVRT), peak isovolumic systolic (IVV), early diastolic (e′), late diastolic (a′), peak systolic (s′) velocity). Myocardial performance index = the sum of isovolumic contraction and relaxation time divided by ejection time (*A*−*B*/*A*)

The MPI, also referred to as Tei index, represents the relation between the sum of isovolumic contraction and relaxation time and ejection time and can be derived from pulsed Doppler or tissue Doppler (Fig. 7). Right ventricular dysfunction (but also increased RV afterload) will increase the time of isovolumic phases and therefore lead to a higher MPI. The index is normally used to estimate global ventricular function of the left ventricle, but the application in the pediatric and neonatal population for right ventricular performance is widely accepted.^45,46^ MPI of both the RV and LV are significantly elevated in infants with PPHN.^25^

Global RV myocardial performance index (MPI) can subsequently be calculated using Eq. 8 .

Reference values for these parameters in the neonatal period have been published recently.^38,42,47^

8 $${\mathrm{MPI}} = \left( {{\mathrm{IVET}} + {\mathrm{IVRT}}} \right){\mathrm{/RVET}} \hfill\\ \left( {{\mathrm{IVET}},{\mathrm{isovolumic}}\,{\mathrm{ejection}}\,{\mathrm{time}};} \right. \hfill \\ {\mathrm{IVRT}},\,{\mathrm{isovolumic}}\,{\mathrm{relaxation}}\,{\mathrm{time}}; \hfill\\ \left. {{\mathrm{RVET}},{\mathrm{right}}\,{\mathrm{ventricular}}\,{\mathrm{ejection}}\,{\mathrm{time}}} \right).$$

#### Right ventricular systolic to diastolic duration ratio (RV S/D ratio)

The right ventricular systolic to diastolic duration ratio (RV S/D ratio) is an index of systolic and diastolic (global) function of the right ventricle and is assumed to reflect ventricular loading and contractility. An increase in S/D ratio is seen as a sign of global right ventricular dysfunction secondary to increased afterload.

**Fig. 8:**
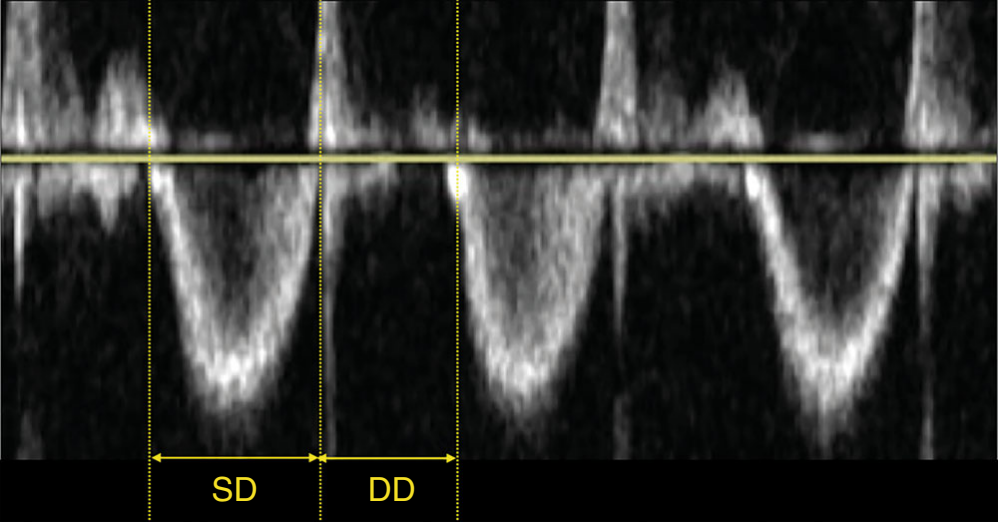
The right ventricular systolic to diastolic duration ratio (RV S/D ratio) is an index of systolic and diastolic (global) function of the right ventricle and is assumed to reflect ventricular loading and contractility (SD systolic duration, DD diastolic duration)

The S/D ratio is calculated from the Doppler signal of tricuspid valve regurgitation. The duration from onset to termination of tricuspid valve regurgitation is the systolic duration (SD) and the diastolic duration is the time between two jets of tricuspid regurgitation (DD), see Fig. 8.^48^ The RV S/D ratio is related to SPAP and RV performance.^24,25,48^ An increased RV S/D ratio (>1.3) is associated with the need for ECMO or death.^25^

#### Tissue Doppler Imaging (TDI)

Diastolic function is traditionally studied by measuring the tricuspid valve inflow velocities (Early (E), Late (A), and ratio (E/A)) during diastole by PW Doppler from the apical 4-chamber view.

TDI derived deformation imaging calculates strain rate by assessing the difference in velocity (the velocity gradient) between two points along the longitudinal plane of the 4-chamber view. Strain is then derived by integrating time into the strain rate values. Only deformation along (parallel to) the beam of the ultrasound is measured by the TD method and is therefore highly dependent on the angle of insonation.

TDI of the right ventricular lateral wall with the sampling gate positioned at the junction of tricuspid annulus allows assessment of systolic and diastolic velocities.

Peak systolic (s′), early and late diastolic (e′ and a′) myocardial velocities can be easily obtained by TDI, including time periods of closing to opening of the tricuspid valve (TcOT′), isovolumic relaxation time (IVRT), and the duration of systole (S) and diastole (D) with the resultant S/D ratio.

Reduced systolic and diastolic TDI velocities have been found in neonates with PH.^49^ In addition, reduced early diastolic velocity on days 1 and 2 of life predicted early respiratory outcome in infants with congenital diaphragmatic hernia.^50^

In term infants with severe PPHN not responsive to iNO, RV strain (−17%) and strain rates (−1.5 1/s) significantly improves following the administration of milrinone over a 24-h period (to −23% and −2.2 1/s, respectively).^51^ This further highlights the ability of deformation parameters to identify myocardial dysfunction and monitor treatment response.

#### Speckle-tracking echocardiography (STE)

STE has been successfully used in the estimation of right ventricular function in term and preterm infants, although the technique was originally developed and validated for the left ventricle of adults.^38,52,53^ As the longitudinal shortening is the main deformation of the right ventricle, longitudinal strain seems to be the most robust parameter in describing systolic right ventricular function.^52^ In addition, diastolic measurements of early and late myocardial movements can be obtained. As an angle-independent method, it does not require geometric assumptions and has been used in the neonatal and preterm population. In term infants with PPHN, a reduced global systolic peak strain of the RV was associated with progression to death or ECMO.^43^ In comparison to healthy controls, term infants with PPHN in the first week of age have worse RV function as shown by a decrease in the magnitude of RV global longitudinal strain.^54^ Similarly, preterm infants with late onset PH (~36 weeks postmenstrual age) also displayed lower values of RV global and free wall longitudinal strain when compared to preterm infants without PH.^55^ Sehgal et al. recently reported the case of a 3-month-old infant where RV function was monitored using STE in response to iNO administration.^56^ A sequential change in global and segmental strain was observed and regional asynchrony in segmental deformation was noted additionally in response to iNO administration. Basal and middle lateral segments showed paradoxical strain (lengthening, positive value). Assessment of regional RV function helped understand adaptive mechanisms and assess therapeutic interventions.

### Ductal and/or atrial shunting

**Fig. 9:**
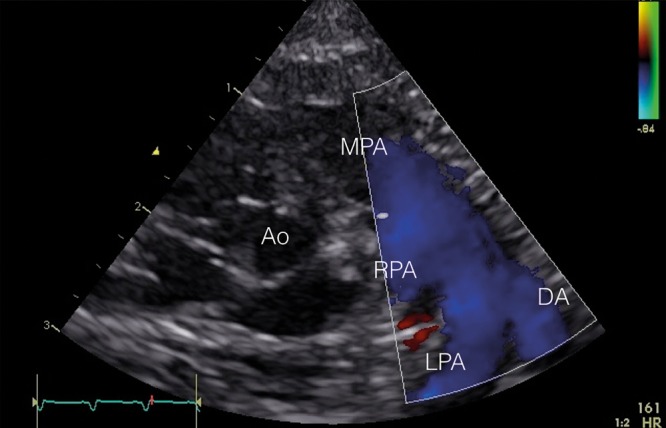
Transductal right-to-left shunting. In the parasternal short axis view, the main pulmonary artery (MPA) is shown with the right pulmonary artery (RPA), the left pulmonary artery (LPA), and the ductus arteriosus (DA) with a clear right-to-left shunt (blue in color Doppler)

Secondary to increased pulmonary arterial pressure, deoxygenated blood can shunt through the fetal channels (ductus arteriosus and foramen ovale) to the systemic circulation leading to hypoxemia (Fig. 9).

In 73–91% of patients with PPHN, a transductal right-to-left or bidirectional shunt can be observed.^21,22,25^ A bidirectional shunt with a systolic right-to-left duration ≥30% of the total heart cycle is considered non-physiologic and likely to represent PPHN. An atrial bidirectional or right-to-left shunt is detected in 73–100% of PPHN patients.^21,22,25^ Also, left-to-right shunting over the interatrial septum is possible, since in PPHN diastolic pulmonary artery pressure is generally sub-systemic with suprasystemic SPAP. A pure right-to-left shunt at the atrial level suggests TAPVC until proven otherwise.

An exclusive right-to-left transductal shunt in patients with PPHN is associated with an increased risk of mortality.^22^ However, this does not imply that shunting is the primary problem; it is merely a marker of disease severity. In the presence of RV dysfunction, shunting through the fetal channels is a mechanism to augment systemic blood flow and offset high RV afterload. The PFO functions as the modulator of (preductal) cerebral blood flow, whereas the PDA modulates blood flow to the body and to some extent to the brain.

## Practical echocardiographic approach to pphn

One of the most important uses of echocardiography in the NICU is in the management of PPHN. Echocardiography should be performed to rule out a congenital heart defect, diagnose PPHN, assess myocardial function, and guide therapy (fluid bolus, choice of cardiovascular drugs). Serial echocardiography is useful in monitoring the response to the treatment in PPHN. We propose a guidance on the assessment of PPHN using NPE, as depicted below. This list is not exhaustive, although not all patients would need all the measurements in the management of PPHN.

1. Structural assessment of the heart to rule out any congenital heart defect, especially duct-dependent heart conditions or significant congenital heart defects; should be done on the first echocardiogram or at the earliest opportunity in a sick neonate.
2. Assessment of (systolic) PAP—this can be measured accurately if there is tricuspid regurgitation (TR). Absence of TR or minimal TR does not rule out PPHN. Assessment of PAP cannot be reliably done via ductal or atrial shunt. However, direction of the shunt will give a good indication about the pressure in relation to the systemic blood pressure.
3. Assessment of the shunt direction across ductus arteriosus—right to left, left to right, or bidirectional
4. Assessment of shunt across foramen ovale—right to left, left to right, or bidirectional. If atrial shunt is purely right to left it should be considered secondary to TAPVC until proven otherwise.
5. Assessment of IVS morphology—flattening or bowing of IVS towards LV suggest supra-systemic PAP or significant volume overloading of the right ventricle (failing right ventricle)
6. Right ventricular size—dilated or hypertrophied or both. RV, RA, and pulmonary artery are commonly enlarged.
7. Objective assessment of RV function (by the methods described in the text—TAPSE, TDI of IVS and RV free wall, PAAT/RVET ratio, RV S/D ratio, RV fractional change, or STE) is recommended. Subjective assessment of RV performance is unreliable and inaccurate.
8. Trend of VTI across pulmonary valve or right ventricular output can be used in monitoring the progress without estimating the cardiac output. The cardiac output is often contaminated by the shunts in this population.
9. Objective assessment of LV function (monitoring myocardial performance (LV-MPI), LV-SV and LVO, ejection fraction (EF biplane Simpson), TDI of IVS and LV free wall, or STE) is recommended.
10. Focused serial echocardiography should be performed to observe the progression, especially in sick infants who are not responding to the intervention.

## Conclusion

PH is a serious disorder that may occur in both term and preterm infants. Neonatologist performed echocardiography is very useful for the timely diagnosis of PH and targeting treatment to prevent morbidity and mortality. For these purposes, many echocardiographic variables can be evaluated, but unfortunately not one of them is the ultimate predictive parameter to assess and manage PH. It is advised to always perform a comprehensive echocardiographic examination. One should bear in mind that NPE will not improve outcome on its own, but it is used to guide treatment and monitor hemodynamic responses that might prove beneficial for the patient’s prognosis.

## APPENDIX

**European Special Interest Group ‘Neonatologist Performed Echocardiography’ (NPE), endorsed by the European Society for Paediatric Research (ESPR) and European Board of Neonatology (EBN)**

**de Boode W. P.** (chairman), Department of Neonatology, Radboud University Medical Center, Radboud Institute for Health Sciences, Amalia Children’s Hospital, Nijmegen, The Netherlands (willem.deboode@radboudumc.nl)

**Austin T.**, Department of Neonatology, Rosie Hospital, Cambridge University Hospitals NHS Foundation Trust, Cambridge, United Kingdom (topun.austin@addenbrookes.nhs.uk)

**Bohlin K.**, Department of Neonatology, Karolinska University Hospital, Karolinska Institutet, Stockholm, Sweden (kajsa.bohlin@ki.se)

**Bravo M. C.**, Department of Neonatology, La Paz University Hospital, Madrid, Spain (mcarmen.bravo@salud.madrid.org)

**Breatnach C. R.**, Department of Neonatology, The Rotunda Hospital, Dublin, Ireland (colm.breatnach@gmail.com)

**Breindahl M.**, Karolinska University Hospital, Karolinska Institutet, Stockholm, Sweden (morten.breindahl@sll.se)

**Dempsey E**, INFANT Centre, Cork University Maternity Hospital, University College Cork, Ireland (g.dempsey@ucc.ie)

**El-Khuffash A.**, Department of Neonatology, The Rotunda Hospital, Dublin, Ireland; Department of Pediatrics, The Royal College of Surgeons in Ireland, Dublin, Ireland (afifelkhuffash@rcsi.ie)

**Groves A. M.**, Division of Newborn Medicine, Mount Sinai Kravis Children’s Hospital, New York, NY, USA (alan.groves@mssm.edu)

**Gupta S.**, University Hospital of North Tees, Durham University, Stockton-on-Tees, United Kingdom (samir.gupta@nth.nhs.uk)

**Horsberg Eriksen B.**, Department of Pediatrics, Møre and Romsdal Hospital Trust, Ålesund, Norway; (beate.eriksen@me.com)

**Levy P. T.**, Department of Pediatrics, Washington University School of Medicine, Saint Louis, MO, USA; Department of Pediatrics, Goryeb Children’s Hospital, Morristown, NJ, USA (Levy_P@kids.wustl.edu)

**McNamara P. J.**, Departments of Pediatrics and Physiology, University of Toronto, Toronto, ON, Canada (patrick.mcnamara@sickkids.ca)

**Molnar Z.**, John Radcliffe Hospital, Oxford, United Kingdom (zoltan.Molnar@ouh.nhs.uk)

**Nestaas E.**, Institute of Clinical Medicine, Faculty of Medicine, University of Oslo, Norway; Department of Cardiology and Center for Cardiological Innovation, Oslo University Hospital, Rikshospitalet, Oslo, Norway; Department of Paediatrics, Vestfold Hospital Trust, Tønsberg, Norway (nestaas@hotmail.com)

**Rogerson S. R.**, The Royal Women's Hospital, Parkville, VIC, Australia (sheryle.Rogerson@thewomens.org.au)

**Roehr C. C.**, Department of Paediatrics, University of Oxford, John Radcliffe Hospital, Oxford, United Kingdom (charles.roehr@paediatrics.ox.ac.uk)

**Savoia M.**, Azienda Ospedaliero-Universitaria S. Maria della Misericordia, Udine, Italy (marilena.savoia@gmail.com)

**Schubert U.**, Department of Clinical Science, Intervention and Technology, Karolinska Institutet, Stockholm, Sweden (ulfschubert@gmx.de)

**Schwarz C. E.**, Department of Neonatology, University Children’s Hospital of Tübingen, Tübingen, Germany (c.schwarz@med.uni-tuebingen.de)

**Sehgal A.**, Department of Pediatrics, Monash University, Melbourne, Australia (arvind.sehgal@monash.edu)

**Singh Y.**, Addenbrooke's Hospital, Cambridge University Hospitals NHS Foundation Trust, Cambridge, United Kingdom (yogen.Singh@nhs.net)

**Slieker M. G.**, Department of Paediatric Cardiology, Radboudumc Amalia Children’s Hospital, Nijmegen, The Netherlands (Martijn.Slieker@radboudumc.nl)

**Tissot C.**, Department of Pediatrics, Clinique des Grangettes, Chêne Bougeries, Switzerland (cecile.tissot@hotmail.com)

**van der Lee R.**, Department of Neonatology, Radboud University Medical Center, Radboud Institute for Health Sciences, Amalia Children’s Hospital, Nijmegen, The Netherlands (Robin.vanderLee@radboudumc.nl)

**van Laere D.**, Department of Pediatrics, Antwerp University Hospital UZA, Edegem, Belgium (david.VanLaere@uza.be)

**van Overmeire B.**, Department of Neonatology, University Hospital Brussels, Brussels, Belgium (bart.van.overmeire@erasme.ulb.ac.be)

**van Wyk L.**, Department of Paediatrics & Child Health, University of Stellenbosch, Cape Town, South Africa (lizelle@sun.ac.za)
